# Human plasma metabolomics in age-related macular degeneration (AMD) using nuclear magnetic resonance spectroscopy

**DOI:** 10.1371/journal.pone.0177749

**Published:** 2017-05-18

**Authors:** Inês Laíns, Daniela Duarte, António S. Barros, Ana Sofia Martins, João Gil, John B. Miller, Marco Marques, Tânia Mesquita, Ivana K. Kim, Maria da Luz Cachulo, Demetrios Vavvas, Isabel M. Carreira, Joaquim N. Murta, Rufino Silva, Joan W. Miller, Deeba Husain, Ana M. Gil

**Affiliations:** 1 Department of Ophthalmology, Massachusetts Eye and Ear, Harvard Medical School, Boston, United States; 2 Faculty of Medicine, University of Coimbra (FMUC), Coimbra, Portugal; 3 Association for Innovation and Biomedical Research on Light and Image (AIBILI), Coimbra, Portugal; 4 Department of Ophthalmology, Centro Hospitalar e Universitário de Coimbra (CHUC), Coimbra, Portugal; 5 CICECO- Aveiro Institute of Materials (CICECO/UA), Department of Chemistry, University of Aveiro, Aveiro, Portugal; University of Manchester, UNITED KINGDOM

## Abstract

**Purpose:**

To differentiate the plasma metabolomic profile of patients with age related macular degeneration (AMD) from that of controls, by Nuclear Magnetic Resonance (NMR) spectroscopy.

**Methods:**

Two cohorts (total of 396 subjects) representative of central Portugal and Boston, USA phenotypes were studied. For each cohort, subjects were grouped according to AMD stage (early, intermediate and late). Multivariate analysis of plasma NMR spectra was performed, followed by signal integration and univariate analysis.

**Results:**

Small changes were detected in the levels of some amino acids, organic acids, dimethyl sulfone and specific lipid moieties, thus providing some biochemical information on the disease. The possible confounding effects of gender, smoking history and age were assessed in each cohort and found to be minimal when compared to that of the disease. A similar observation was noted in relation to age-related comorbidities. Furthermore, partially distinct putative AMD metabolite fingerprints were noted for the two cohorts studied, reflecting the importance of nutritional and other lifestyle habits in determining AMD metabolic response and potential biomarker fingerprints. Notably, some of the metabolite changes detected were noted as potentially differentiating controls from patients diagnosed with early AMD.

**Conclusion:**

For the first time, this study showed metabolite changes in the plasma of patients with AMD as compared to controls, using NMR. Geographical origins were seen to affect AMD patients´ metabolic profile and some metabolites were found to be valuable in potentially differentiating controls from early stage AMD patients. Metabolomics has the potential of identifying biomarkers for AMD, and further work in this area is warranted.

## Introduction

Age-related Macular Degeneration (AMD) is the leading cause of adult blindness in developed countries and the third most common cause of adult blindness worldwide. It is anticipated that global AMD prevalence will reach 196 million in 2020 and 288 million in 2040.[1] Approximately 90% of patients with AMD have early or intermediate forms, which may progress to advanced disease, in the form of either geographic atrophy and/or neovascular AMD (also known as “wet AMD”).[2],[3] Often asymptomatic in the early stages, in some patients AMD ultimately leads to loss of central vision deterioration and interferes with daily living activities, with profound effects on the quality of life of the elderly.[4] AMD pathogenesis is multifactorial, with complex genetic risk factors interacting with several lifestyle and environmental factors.[2],[3] Serologic biomarkers of AMD incidence and progression have been sought, primarily relating to pathways responsive to inflammation,[5–7] cell stress (particularly oxidative stress) or toxicity.[2],[7],[8] Such studies have, however, provided inconsistent results and, hence, clinical practice still solely relies on the evaluation of phenotypic characteristics, such as fundus appearance. Biofluid markers may be useful to help predict incidence and prevalence of the disease, but reliable markers are still lacking.

Metabolomics, the qualitative and quantitative analysis of metabolites (M_w_ <1 kDa),[9] may provide an integrated biomarker signal for AMD. Metabolites are the downstream result of genetic transcription processes and, simultaneously, reflect environmental and lifestyle factors as well as individual characteristics related to dietary response and gut microflora.[10],[11] The metabolome should therefore closely reflect the global health state and phenotype of the subject. Two complementary analytical techniques are currently used in metabolomics: mass spectrometry (MS) and nuclear magnetic resonance (NMR) spectroscopy. Briefly, MS provides higher sensitivity, and NMR offers higher reproducibility, simpler sample handling and the possibility of sample reuse.[12],[13] The potential of metabolomics has been established in the search for biofluid (blood and urine) marker profiles for several diseases, including cancer.[14–16] In ophthalmology, recent reviews highlighted its potential applications,[17–19] although results are still scarce and mostly related to animal models.[19] Metabolomic studies of human samples using liquid chromatography (LC)-MS analysis of tears of healthy subjects[20] and patients with keratoconus suggested differences in metabolites related to the urea cycle, Krebs’ cycle and oxidative stress.[21] Additionally, NMR studies of vitreous samples from subjects with proliferative vitreoretinopathy and retinal detachment suggested that these diseases might present a specific metabolic signature.[22],[23],[24]. Metabolomics of human plasma has been used to characterize diabetic retinopathy[25],[26], open angle glaucoma[27] and anterior uveitis.[28] In AMD, investigators have used untargeted LC-MS metabolomics to investigate changes in the plasma of “wet” AMD patients (n = 26), compared to controls (n = 19).[29]

In the current study, we have used NMR metabolomics to characterize the plasma metabolomic signatures of patients with AMD at different severity stages (early, intermediate and late AMD stages), considering two large cohorts from distinct geographical origins, Southern European (Coimbra, central Portugal) and Northeastern United States (Boston Metropolitan area, US), which allows for evaluation of geographical effects on metabolomic profiles. We further investigated signature specificity to AMD by evaluating the impact of potential confounders (gender, smoking history, body mass index (BMI) and age) on plasma profile. The strong age dependence of AMD presents a significant challenge in the search for disease-specific markers. Due to the difficulty in using age-matched groups in this context, we have performed an objective evaluation of the impact of age on metabolic profile and used unmatched cohorts, which better represent actual AMD patients’ population. The potential confounding role of comorbidities is also discussed.

## Materials and methods

### Study design and subject recruitment

This study is part of a cross-sectional, observational study performed in the Department of Ophthalmology of Massachusetts Eye and Ear (MEE), Harvard Medical School, Boston, United States, and the Faculty of Medicine of the University of Coimbra (FMUC), Coimbra, Portugal, in collaboration with the Association for Innovation and Biomedical Research on Light and Image (AIBILI) and the “Centro Hospitalar e Universitário de Coimbra”, Coimbra, Portugal. The clinical protocol was conducted in accordance with HIPAA (Health Insurance Portability and Accountability Act) requirements and the tenets of the Declaration of Helsinki, and was approved by the Institutional Review Boards of MEE, FMUC and AIBILI, and by the Portuguese National Data Protection Committee (CNPD). All subjects enrolled in the study provided written informed consent.

From January 2015 to July 2016, in both sites (Coimbra and Boston), patients diagnosed with AMD were prospectively recruited, as well as control groups of subjects with no evidence of AMD and aged ≥ 50 years. At MEE, participants were consecutively recruited from the Retina Service and the Comprehensive Ophthalmology and Optometry Services, at their regular appointments. For those not fasting at that time, a new appointment was scheduled within a maximum of one month for blood collection under fasting. The Portuguese (FMUC/AIBILI) study population was derived from a population-based cohort study,[30] where all subjects with an established diagnosis of any stage of AMD were invited to participate. Subjects without signs of AMD in a prior evaluation[30] were also invited, and included as controls if they remained without the disease in the present evaluation (see criteria below). For both cohorts, the exclusion criteria were: diagnosis of any other vitreoretinal disease, active uveitis or ocular infection, significant media opacities that precluded the observation of the ocular fundus, refractive error equal or greater than 6 diopters of spherical equivalent, past history of retinal surgery, history of any ocular surgery or intra-ocular procedure (such as laser and intra-ocular injections) within the 90 days prior to enrolment, and diagnosis of diabetes mellitus. Other common age-related conditions (hypertension, dyslipidemia, rheumatic disease, renal or liver conditions, and neurological diseases) were not considered for exclusion and their potential effect on the results will be discussed.

### Clinical examination

All participants received complete bilateral ophthalmologic examination, including a dilated fundus exam. Recruited subjects were also imaged with 7-field, non-stereoscopic color fundus photographs (CFP) either with a Topcon TRC-50DX (Topcon Corporation, Tokyo, Japan) or a Zeiss FF-450Plus (Carl Zeiss Meditec, Dublin, CA) camera. At the same visit, a complete medical history was obtained, according to a standardized questionnaire specifically built for the purposes of this study (S1 Appendix), based on the current knowledge of AMD pathogenesis and the input of five Retina specialists. This included self-reported data on smoking habits (smokers were considered those who reported current smoking and ex-smokers those who have ever smoked, regardless of when they stopped), and weight and height (used for BMI calculations). If the study participants did not know their current height and/or weight, these were recorded by a study investigator.

For AMD diagnosis and staging, two of three independent experienced graders analyzed all field 2 CFP, according to the AREDS classification system.[31]^,^[32] In case of disagreement, a senior author (RS or DH) established the final categorization. Images taken with Topcon cameras were evaluated with IMAGEnet 2000 software (version 2.56; Topcon Medical Systems), and those obtained with a Zeiss camera were observed using VISUPAC (version 4.5.1; Carl Zeiss Meditec). Images were standardized using software developed by our group. We adopted the most recent AREDS2 definitions,[32] namely that the standard disc diameter equals 1800 μm (rather than 1500 μm), which affects the size of the ETDRS grid and of the standard drusen circles; and that geographic atrophy (GA) is present if the lesion has a diameter equal or superior than 433 μm (AREDS circle I-2) and at least two of the following features are present—absence of RPE pigment, circular shape, or sharp margins (thus meaning that the involvement of the central fovea is not a requirement). Therefore, the following groups were established and used for further assessments[31],[32]: controls–presence of drusen maximum size < circle C0 and total area < C1; early AMD–drusen maximum size ≥ C0 but < C1 or presence of AMD characteristic pigment abnormalities in the inner or central subfields; intermediate AMD–presence drusen maximum size ≥ C1 or of drusen maximum size ≥ C0 if the total area occupied is > I2 for soft indistinct drusen and > O2 for soft distinct drusen; late AMD–presence of GA according to the criteria described above (GA or “dry” late AMD) or evidence of neovascular AMD (choroidal neovascularization, CNV or “wet” AMD). For participants with different stages in the two eyes, the worse of the two was considered as the subjects’ classification.

### Plasma collection and NMR analysis

The present cross-sectional study relied on a single plasma collection per individual. For all participants, fasting blood samples were collected into sodium-heparin tubes, and centrifuged within 30 min (1500 rpm, 10 min, 20°C). Plasma aliquots of 1.5 mL (MEE) and 5 mL (FMUC/AIBILI) were transferred into sterile cryovials and stored at -80°C. Plasma samples from MEE were shipped to Portugal for metabolomic profiling in dry ice (through TNT Express, USA, INC). Samples arrived frozen in less than 48 hours and were immediately stored at -80°C until NMR analysis. Prior to analysis, plasma samples were thawed at room temperature and homogenized in a vortex mixer. Then, 400 μl of saline solution (NaCl 0.9% in 10% D_2_O) were added to 200 μl of sample. After centrifugation (4500 g, 5min, 25°C), 550 μl of each sample was transferred to a 5 mm NMR tube. NMR spectra were recorded, at 300 K, using a Bruker Avance DRX 500 spectrometer operating at 500.13 MHz for proton, with a 5 mm TXI probe. For each sample, three one-dimensional (1D) ^1^H NMR spectra were obtained: a standard spectrum (*noesypr1d*, Bruker library), a Carr−Purcell−Meiboom−Gill (CPMG) spectrum (*cpmgpr*) and a diffusion-edited spectrum (*ledbpgp2s1dpr*). Standard spectra were acquired with a 100 ms mixing time and water suppression during the relaxation delay (RD = 4 s) and mixing time. CPMG spectra were acquired with water presaturation, with 80 loops (n) and a total spin−spin relaxation time (2nτ) of 64 ms (with τ = 400 μs). Diffusion-edited spectra were recorded with a diffusion time of 100 ms, a pulsed-field gradient (G1) of 1 ms, a spoil gradient (G2) of 0.6 ms, an eddy current recovery time (τ) of 5 ms, and 90% of the maximum gradient strength (48.15 G/cm). All 1D spectra were acquired into 32k complex data points with 10330.58 Hz spectral width. Each free induction decay was zero-filled to 64 k points and multiplied by a 0.3 Hz exponential line-broadening function prior to Fourier transformation. Spectra were manually phased and baseline corrected and chemical shifts referenced internally to the α-glucose H1 resonance (δ 5.23). Peak assignments were carried out using 2D NMR spectra and databases Bruker B-BIOREFCODE and HMDB.[33]

### Statistical analysis of metadata and NMR data

Statistical descriptive and inference methods (t-test, Fisher-exact test, Chi-square test and density distribution) were used to describe the clinical and demographic characteristics of the study population. Multivariate analysis was applied to a total of 729 NMR spectra from Coimbra cohort (42 controls and 201 AMD patients) and 459 spectra from Boston cohort (40 controls and 113 AMD patients). For each type of spectrum (standard, CPMG, diffusion-edited), data matrices were built (Amix 3.9.14, Bruker, BioSpin, Rheinstetten, Germany) excluding the water region (δ 4.50–5.15). Spectra were aligned using recursive segment-wise peak (RSPA),[34] normalized to total area, and scaled to unit variance. Initial analysis by Principal Component Analysis (PCA),[35] an unsupervised technique (no consideration of sample class or characteristics) that accommodates all inter-subject variability sources, was followed by supervised assessments (where information on sample class is considered) by Partial Least Squares Discriminant Analysis (PLS-DA),[36] and orthogonal (O)-PLS-DA[37] (SIMCA-P 11.5, Umetrics, Umeå, Sweden). PLS-DA loading weights were back-transformed, multiplying each variable by its standard deviation, and false-colored according to variable importance to the projection (VIP) (Matlab 7.9.0, The MathWorks, Inc, Natick, MA). For PLS-DA models, randomized (Monte Carlo) cross validation (MCCV, in-house developed) was carried out, with recovery of Q^2^ values and confusion matrices; simultaneous randomized class-permutation assessed the null hypothesis. Classification rates (CR), specificity (spec.), and sensitivity (sens.) were computed.

In order to filter out random phenotypic effects unrelated to AMD, variable selection was employed (with VIP > 1, VIP/VIPcvSE > 1 and |b/bcvSE| > 1)[38] and PLS-DA reapplied. The models with higher Q^2^ values enabled relevant resonances to be identified and subsequently integrated across AMD stages (Amix 3.9.14). Integrals were normalized to total intensity and variations assessed by univariate analysis (Wilcoxon test, R statistical software). Effect size and corresponding confidence intervals were computed using the Hedges’ g index.[39] Generalized linear regression was used as a flexible generalization of ordinary multiple linear regression by allowing the magnitude of the variance of each measurement (age, sex, BMI, and AMD status) to be a function of the metabolite semi-quantitative variation.

## Results

### Study population

We recruited a total of 396 subjects, 61% (n = 243) in Coimbra (42 controls and 201 patients with AMD) and the remaining in Boston (n = 153) (40 controls and 113 patients with AMD). The study population demographics and AMD staging classification are shown in Table 1. Not surprisingly, there were significant differences in age (*p*-values 1.3x10^-2^–3.2x10^-5^) and age distributions (S1 Fig, left) between controls and AMD patients in both cohorts and across the stages of AMD, except between controls and early AMD (*p*-values 0.31 and 0.55, for Coimbra and Boston, respectively). This confirms age as a potentially important confounder in human plasma profiling studies of AMD. Similarly, the possible confounding nature of gender among AMD severity groups (in Coimbra only, *p*-value 4.2x10^-2^) and smoking history (expressed as ex-smokers/non-smokers ratio, as only residual numbers of smokers were identified) was also considered. No significant BMI differences were observed across groups (*p-*values > 0.2) (Table 1 and S1 Fig, right). Furthermore, the age-related comorbidities observed in our cohorts were well balanced (S2 Table), except for a small number of conditions between intermediate and late AMD groups.

**Table 1:** Characterization of the study population. Characterization of the study populations (Coimbra and Boston cohorts), with corresponding number of subjects (n), age (years), female (F)/male (M) ratio, body mass index (BMI) (kg.m^-2^) and smoking history.

	Coimbra cohort (n_total_ = 243)				Boston cohort (n_total_ = 153)			
	Controls	Early AMD	Int. AMD	Late AMD	Controls	Early AMD	Int. AMD	Late AMD
n ^a^	42 (17.3)	45 (18.5)	124 (51.0)	32 (13.2) ^b^	40 (26.1)	30 (19.6)	45 (29.4)	38 (24.8) ^c^
Age (years)	68 (58–77)	70 (61–82)	75 (60–91)	81 (62–92)	70 (51–95)	68 (54–91)	71 (61–85)	75 (56–89)
Gender (F/M)	26/16	29/16	85/39	16/16	24/16	20/10	29/16	24/14
BMI (kg.m^-2^) ^d^	27 (18–38)	27 (18–36)	28 (19–42)	27 (17–36)	26 (19–40)	26 (18–39)	27 (21–53)	26 (20–37)
Smoking history ^e^:								
Smokers	0	0	0	1	2	0	2	0
ex-smokers	8	6	14	11	16	11	24	23
non-smokers	34	39	110	20	21	19	19	13

Int. AMD: intermediate AMD.

^a^: numbers in brackets correspond to % of cohort

^b^: further classified as “wet” (n = 27) and “dry” AMD (n = 5)

^c^: further classified as “wet” (n = 31) and “dry” AMD (n = 7)

^d^: information unavailable for 2 Coimbra subjects (1 early and 1 intermediate AMD) and 14 Boston subjects (3 controls and 2 early, 4 intermediate and 5 late AMD)

^e^: information unavailable for 3 Boston subjects (1 control and 2 late AMD).

### Plasma profile differences

**Fig 1:**
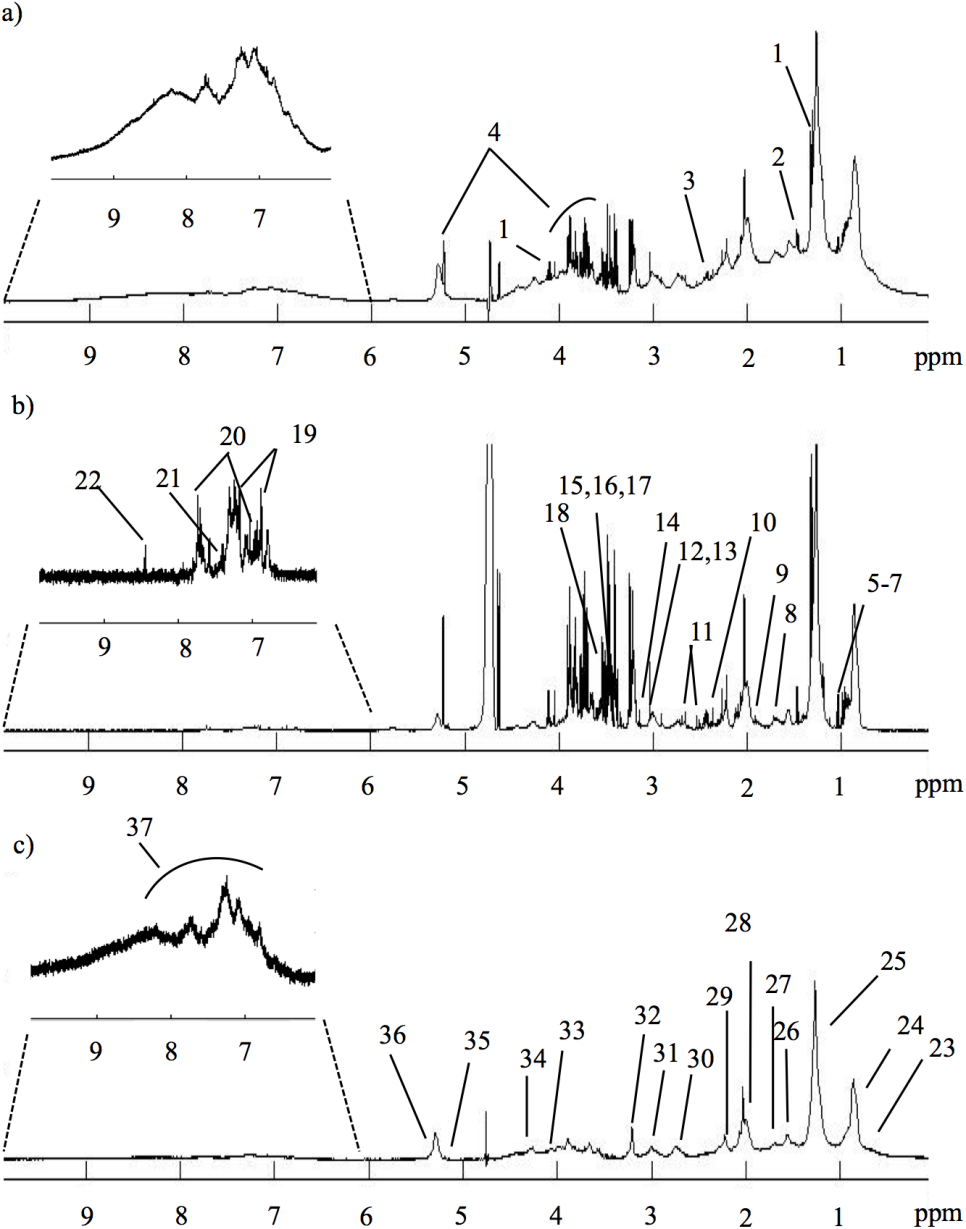
Representative ^1^H NMR spectra of control plasma. 500 MHz ^1^H NMR spectra of blood plasma from a control subject: a) standard 1D spectrum; b) CPMG spectrum; c) diffusion-edited spectrum. Signal assignment: 1-lactate; 2-alanine; 3 -glutamine; 4-glucose; 5-isoleucine; 6-leucine; 7-valine; 8-lysine; 9-acetate; 10-pyruvate; 11-citrate; 12-creatine; 13-creatinine; 14-dimethyl sulfone; 15-TMAO, trimethylamine-*N*-Oxide; 16,proline; 17-methanol; 18-glycine; 19-tyrosine; 20-histidine; 21- phenylalanine; 22-formate; 23-C18H cholesterol; 24-CH_3_ lipids; 25-(CH_2_)_n_ lipids; 26-CH_2_CH_2_CO lipids; 27-CH_2_CH_2_C = C lipids; 28-CH_2_C = C lipids; 29-CH_2_CO lipids; 30-C = CCH_2_CH = C lipids; 31-albumin lysil groups; 32-N(CH_3_)_3_ choline; 33-glyceryl C1,3H; 34-glyceryl C1,3H’; 35-glyceryl C2H; 36-HC = CH lipids; 37-NH protein region.

Fig 1 shows representative standard, CPMG and diffusion-edited ^1^H NMR spectra of a control plasma sample, reflecting all visible compounds (standard spectrum, Fig 1A), mainly low-M_w_ compounds (CPMG spectrum, Fig 1B) and high-Mw compounds (diffusion-edited spectrum, Fig 1C), respectively. Editing of spectral information in the latter two spectra (Fig 1B and 1C) reduces spectral overlap and provides specific information on small molecule and macromolecule metabolomes, respectively. It should be noted, however, that lipid resonances corresponding to smaller and/or more mobile lipids also contribute to CPMG spectra. Overall, spectral assignment revealed just under 30 low-M_w_ metabolites, in agreement with previous reports,[40] and several different macromolecule environments arising from proteins (albumin, glycoproteins) and lipoprotein cholesterol, choline, glyceryl and fatty acid moieties (lipids assignment shown in S1 Table).

Initial PCA revealed no separation between controls and AMD patients in either cohort, thus reflecting high inter-individual variability of plasma profiles. Subsequent pairwise PLS-DA analysis of full CPMG and diffusion-edited spectra provided models with no classification power (Q^2^ < 0.5) for AMD status in either cohort (S3 Table). The highest pairwise model Q^2^ values were observed in the Boston cohort for: intermediate *vs*. early AMD (CPMG spectra, i.e. low-M_w_ metabolites domain) (Q^2^ = 0.32, 80% sensitivity, 53% specificity); and late *vs* early AMD (diffusion-edited spectra or high M_w_ domain) (Q^2^ = 0.39, 78% sensitivity, 79% specificity) (S3 Table). This indicates that AMD does not strongly impact the plasma profile when analyzed by NMR (i.e. when metabolites down to sub-millimolar concentrations are considered). Furthermore, no distinction was detected between the small sub-cohorts of late AMD subjects with “dry” (geographic atrophy) or “wet” (choroidal neovascularization) AMD (^b^ and ^c^ in Table 1).

**Fig 2:**
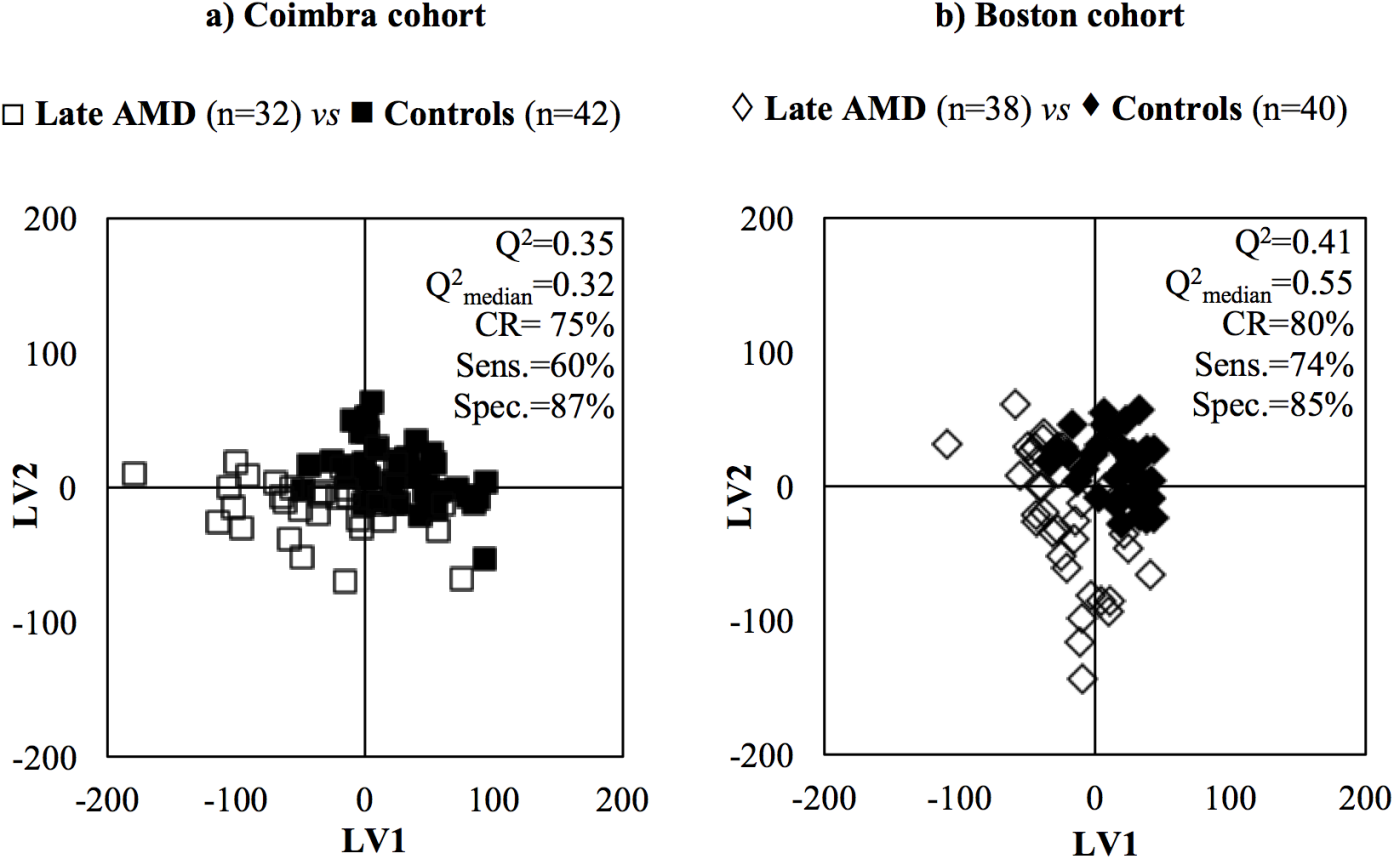
Examples of PLS-DA score plots. PLS-DA scores scatter plots and MCCV quality parameters (pairwise model Q^2^, Q^2^_median_ (obtained through MCCV), % CR, % sens. and % spec.) obtained for variable selected CPMG NMR spectra of late AMD patients *vs* controls, in the a) Coimbra cohort: late AMD patients (□, n = 32), controls (∎, n = 42) and b) Boston cohort: late AMD patients (◇, n = 38), controls (♦, n = 40).

**Fig 3:**
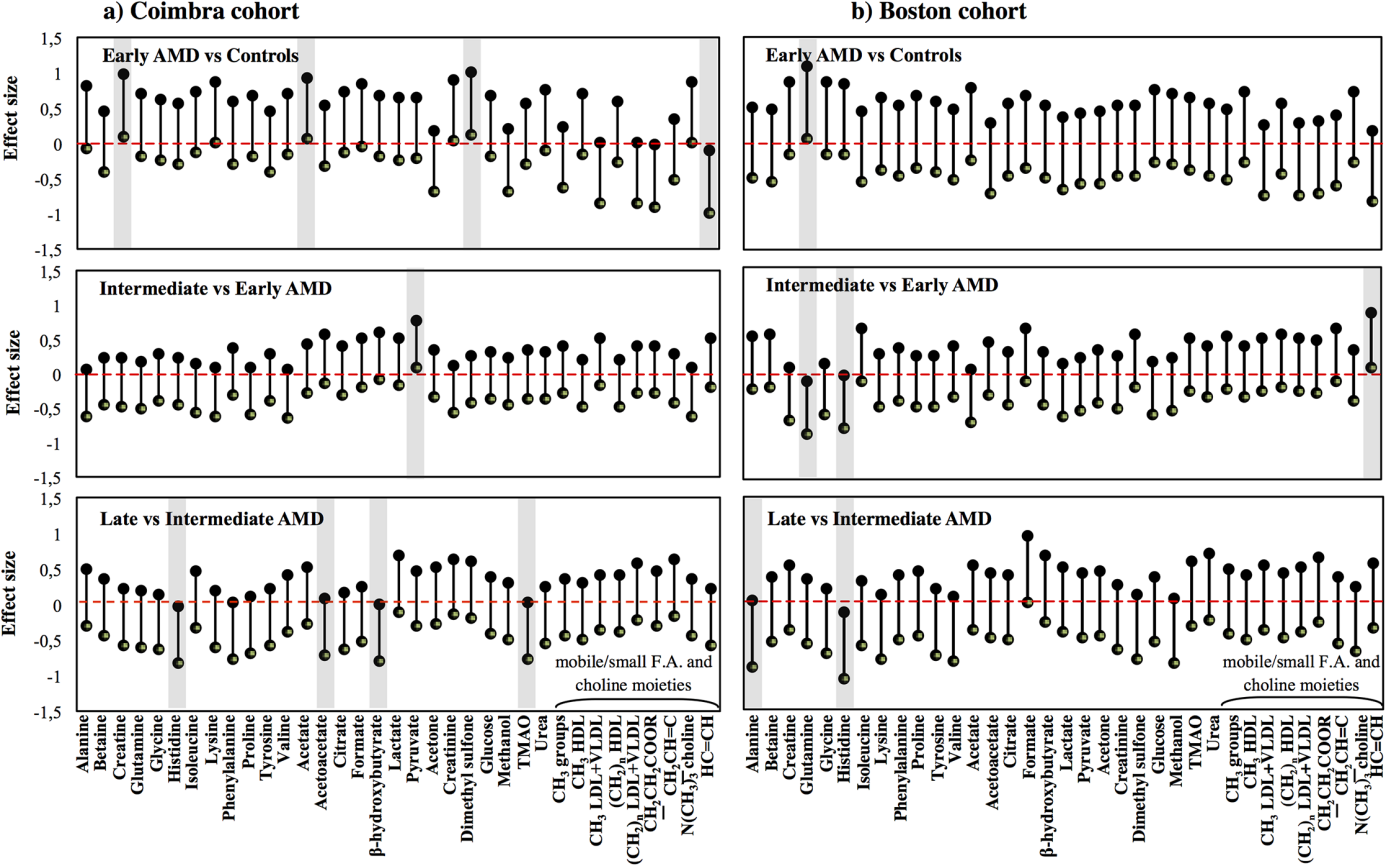
Effect size plots for CPMG spectra integrals. Effect size (E.S.) plots for resonances varying in the CPMG NMR spectra across AMD evolution through different severity stages in the a) Coimbra and b) Boston cohorts. Resonances are listed alphabetically within each compound family (amino acids, organic acids, other low-M_w_ compounds and lipids). The dashed horizontal line refers to null E.S. and the length of the vertical segments corresponds to E.S. range. E.S. segments not intercepting the null E.S. line are considered as relevant variations (shaded rectangles). F.A.: fatty acids.

**Fig 4:**
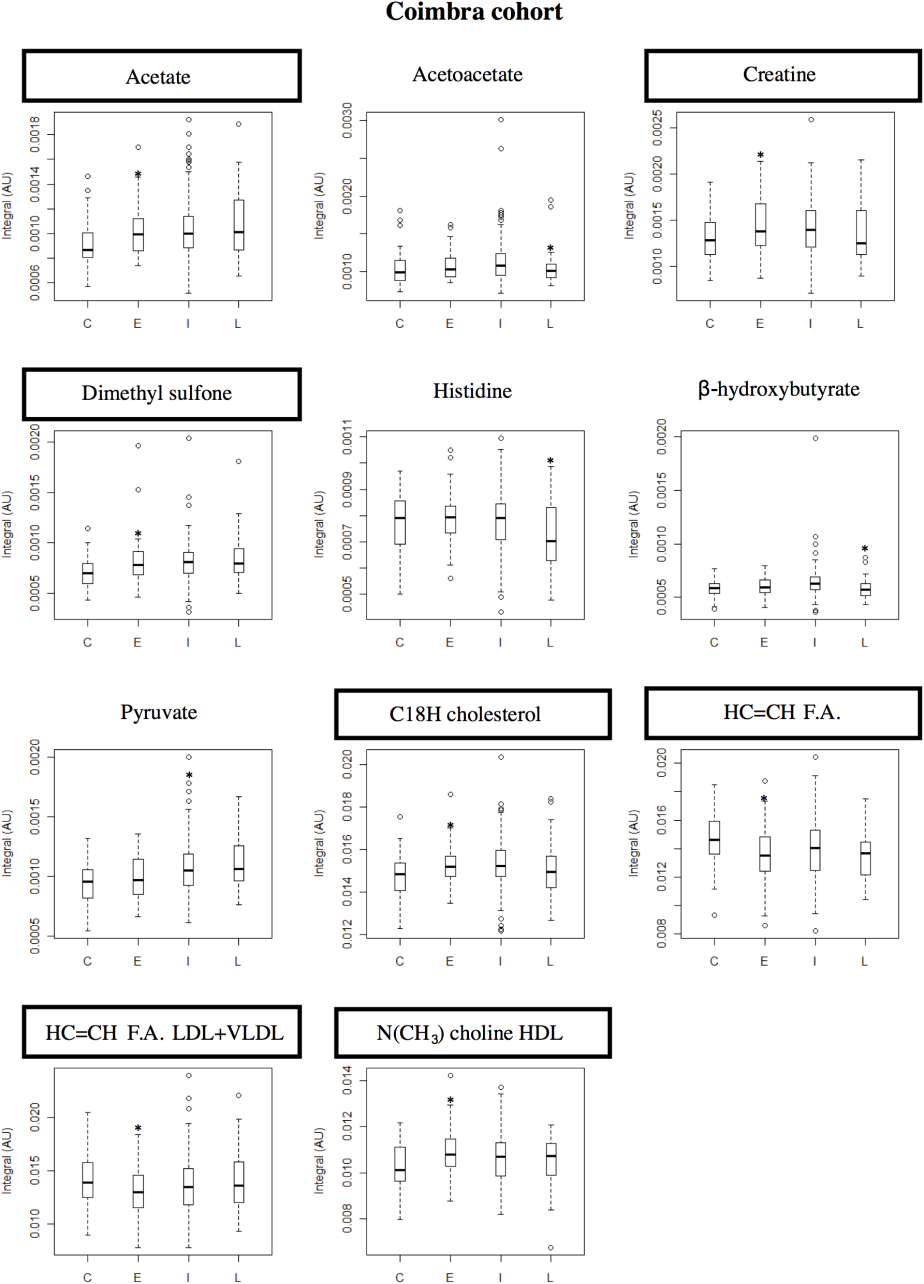
Boxplot graphs for metabolites varying in Coimbra cohort. Coimbra cohort: boxplot representations of the metabolite variations found statistically relevant (* indicates *p-*value < 0.05) in at least one pairwise PLS-DA model. Compound names in rectangles correspond to compounds differentiating between controls and early AMD patients. C: controls, E: early AMD, I: intermediate AMD, L: late AMD. F.A.: fatty acids.

**Fig 5:**
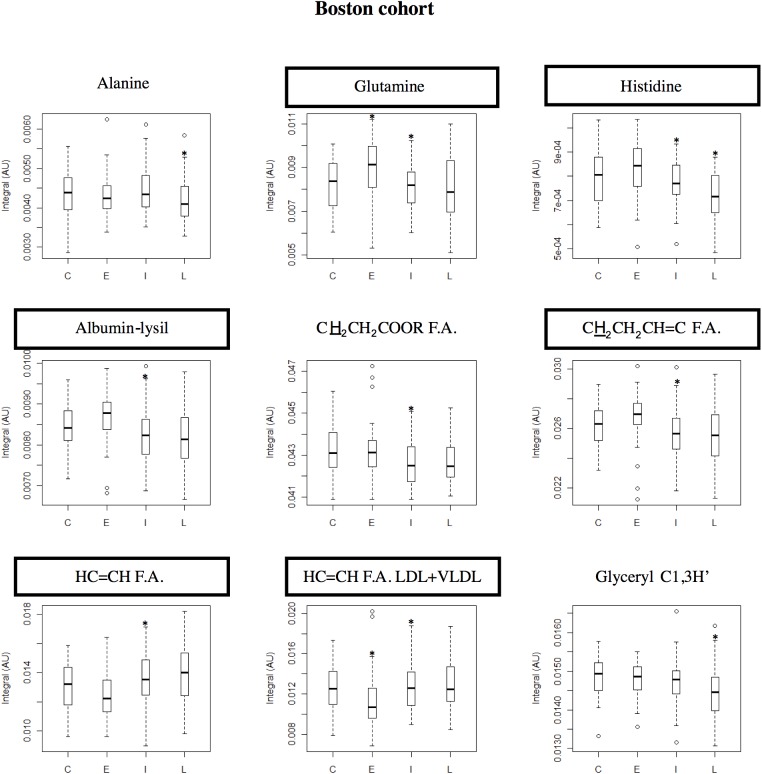
Boxplot graphs for metabolites varying in Boston cohort. Boston cohort: boxplot representations of the metabolite variations found statistically relevant (* indicates *p-*value < 0.05) in at least one pairwise PLS-DA model. Compound names in rectangles correspond to compounds differentiating between controls and early AMD patients. C: controls, E: early AMD, I: intermediate AMD, L: late AMD. F.A.: fatty acids.

Variable selection was then performed to filter off random variability unrelated to sample classes.[38] In this study, it led to improved model robustness for some pairwise comparisons, in both cohorts (as shown by higher values of Q^2^, CR, sensitivity and specificity, underlined bold in S3 Table), and some group separation in PLS-DA score plots. Fig 2 shows the PLS-DA score plots obtained when comparing late AMD subjects with controls. In the Coimbra cohort, group separations were mostly seen between extreme stages (late AMD *vs* controls and late *vs* early AMD, with Q^2^ = 0.32–0.35). In Boston, group separations were also noted between multiple AMD stages; for example, for early AMD *vs* controls, Q^2^ = 0.50–0.54 (S3 Table). The best performing models were further studied through analysis of the corresponding loading plots, signal integration and subsequent calculation of effect size (E.S.). The metabolites changing significantly in at least one of the pairwise models in S3 Table were thus identified, and tracked thereafter across the various degrees of AMD severity (Table 2). The graphical representations of E.S. for low-M_w_ and macromolecule metabolomes (Fig 3 and S2 Fig, respectively) help visualize the most relevant metabolite variations across AMD stages (Table 2), as do the boxplot representations in Figs 4 and 5.

**Table 2:** Variations in plasma metabolites of AMD patients. Main variations in plasma metabolites across AMD evolution through different severity stages, in Coimbra and Boston cohorts.

Coimbra cohort				Boston cohort			
Family	Compound (δ/ppm, multiplicity)	E.S.	*p-*value^a^	Family	Compound (δ/ppm, multiplicity)	E.S.	*p-*value^a^
**Early AMD *vs* Controls**							
A.A.	Creatine (3.03, s)^b^	0.53 [0.09,0.97]	2.9E-2	A.A.	Glutamine (2.43, m)^b^	0.59 [0.07,1.10]	1.6E-2
Lipids	C18H cholesterol (0.59–0.70)^c^	0.52 [0.08,0.96]	2.1E-2	Lipids	HC = CH F.A. LDL+VLDL (5.28–5.37)^c^	-0.36 [-0.85, 0.13]	2.9E-2
	HC = CH F.A. (5.24–5.37)^b^	-0.55 [-0.99,-0.11]	1.6E-2					
	N(CH_3_)_3_ choline HDL (3.16–3.21)^c^	0.65 [0.21,1.10]	4.9E-3					
	HC = CH F.A. LDL+VLDL (5.28–5.37)^c^	-0.50 [-0.94,-0.06]	2.9E-2					
O.A.	Acetate (1.91, s)^b^	0.50 [0.06,0.94]	1.6E-2				
Other	Dimethyl sulfone (3.15, s)^b^^,^ ^d^	0.57 [0.13,1.01]	7.1E-3				
**Intermediate *vs* Early AMD**							
O.A.	Pyruvate (2.36, s)^b^	0.43 [0.08, 0.78]	1.8E-2	A.A.	Glutamine (2.43, m)^b^ Histidine (7.74, s)^b^	-0.65 [-1.15,-0.15] -0.54 [-1.04, -0.04]	6.4E-3 1.9E-2
				Lipids	CH_2_CH_2_COOR F.A. (1.45–1.61)^c^	-0.57 [-1.06, -0.09]	3.6E-2
					CH_2_CH_2_C = C F.A. (1.62–1.74)^c^	-0.57 [-1.05,-0.08]	2.3E-3
					HC = CH F.A. (5.24–537)^b^	0.63 [0.13,1.13]	6.1E-3
					HC = CH F.A. LDL + VLDL (5.28–5.37)^c^	0.46 [-0.02,0.95]	7.4E-3
			
				Other	Albumin lysil (2.92–3.02)^c^	-0.56 [-1.04,-0.07]	4.5E-3
**Late *vs* Intermediate AMD**							
A.A.	Histidine (7.74, s)^b^	-0.43 [-0.82,-0.03]	4.0E-2	A.A.	Alanine (1.47, d)^b^	-0.44 [-0.90,0.03]	4.2E-2
					Histidine (7.74, s)^b^	-0.61 [-1.07,-0.14]	1.3E-2
O.A.	Acetoacetate (2.27, s)^b^	-0.31 [-0.70, 0.09]	3.7E-2				
	β-hydroxybutyrate (2.39, m)^b^	-0.40 [-0.80,-0.005]	1.0E-3	Lipids	Glyceryl C1,3H’(4.20–4.34)^c^	-0.43 [-0.87,0.02]	3.0E-2

E.S.: effect size, values in square brackets correspond to E.S. range; A.A.: amino acids, O.A.: organic acids, d: doublet, s: singlet, m: multiplet, F.A.: fatty acids.

^a^: all p-values indicated become > 0.05 upon Bonferroni correction for multiple comparisons.

^b^ and ^c^: integrals measured in CPMG and in diffusion-edited spectra, respectively

^d^: metabolite with possible contribution from different subjects’ age.

Compared to controls, Coimbra subjects with early AMD exhibited higher circulating levels of creatine, acetate and dimethyl sulfone (Table 2, shaded areas in Fig 3A, top), C18 cholesterol and HDL-choline resonances (S2A Fig, top); and lower levels of unsaturated fatty acids (F.A.) (Table 2, Fig 3A and S2A Fig, top). In this cohort, increasing AMD severity produced small changes in low-M_w_ metabolites (Table 2, Fig 3A): higher pyruvate for intermediate AMD and lower levels of histidine, acetoacetate, β-hydroxybutyrate and trimethylamine-*N*-oxide (TMAO) for late AMD (the latter not statistically relevant). In the macromolecules domain (S2A Fig), qualitative lower protein levels (expressed by NH resonances) were noted in intermediate AMD and initial low levels of unsaturated F.A. increased at later AMD stages. Boston subjects differed slightly in terms of the low-M_w_ domain (Table 2, Fig 3B), exhibiting higher levels of glutamine in early AMD and lower levels for intermediate AMD; low histidine levels in intermediate and late AMD (similarly to the Coimbra cohort); and low alanine levels in late AMD. In the macromolecule domain (S2B Fig), results were broadly similar between cohorts, with unsaturated fatty acid levels again increasing from early AMD into more severe stages, and a tendency for low protein levels (including albumin) characterizing intermediate AMD.

In the boxplot representations for Coimbra (Fig 4) and Boston (Fig 5) cohorts, a number of small metabolite variations (some only qualitative in nature) emerge as potentially differentiating controls from early AMD, namely: 1) acetate, creatine, dimethyl sulfone, cholesterol, HDL-choline and unsaturated fatty acids in Coimbra subjects and 2) glutamine, histidine, unsaturated fatty acids and albumin, in Boston subjects (highlighted metabolite names in Figs 4 and 5). This observation refers to the two naturally age-matched groups of controls and early AMD, thus not being affected by possible age confounding effects, and may potentially lay the ground for future AMD biomarkers. The role of age and other characteristics as possible confounders is discussed in the next section.

### Potential confounders

We evaluated the importance of age and other potential confounders, namely gender and smoking history (as mentioned, BMI was not statistically different in these cohorts), on the subjects´ plasma metabolic profile and, in particular, on the putative AMD fingerprints shown in Table 2. To investigate this, the larger groups of subjects diagnosed with intermediate AMD (Table 1) were used for multivariate analysis as a function of each parameter, independently of AMD staging. Regarding the gender imbalance between intermediate and late AMD groups in Coimbra (no imbalance was observed in Boston), PLS-DA indicated that Coimbra females had lower circulating levels of isoleucine (*p-*value = 8.85x10^-6^), valine (*p-*value = 2.13x10^-5^), creatinine (*p-*value = 4.74x10^-5^), lactate (*p-*value = 2.09x10^-4^) and methanol (*p-*value = 1.67x10^-3^). Assuming that such differences are independent of AMD staging, their absence in the putative AMD fingerprint in Table 2 indicates that gender is not a confounder in this cohort. Regarding smoking habits, ex-smokers in Coimbra were found to have slightly higher levels of formate (*p-*value = 5.65x10^-3^) than non-smokers; increases in methanol (*p-*value = 2.96x10^-2^) and lactate (*p-*value = 2.57x10^-2^) were also noted but probably are due to a higher proportion of female ex-smokers in this group. In Boston, ex-smokers showed a slight increase in isoleucine (*p-*value = 4.35x10^-2^), compared to non-smokers. Since none of these changes coincided with the suggested AMD fingerprints (Table 2), smoking history may also be ruled out as an important metabolic confounder in these cohorts. Finally, PLS-DA models of age of intermediate AMD groups revealed increases in acetoacetate (*p-*value = 3.8x10^-2^), citrate (*p-*value = 1.9x10^-4^), dimethyl sulfone (*p-*value = 1.4x10^-2^) and β-hydroxybutyrate (*p-*value = 8.6x10^-3^) in older Coimbra subjects, whereas older Boston subjects exhibited a weak tendency for higher circulating urea levels (*p-*value = 3.9x10^-2^). Again, these variations do not compromise the potential fingerprint features, except for dimethyl sulfone (^c^ in Table 2, note the opposite variation of β-hydroxybutyrate which rules it out as originating from subjects´ older age). Dimethyl sulfone was only seen in the Coimbra cohort, and its variation differentiated groups with similar age (controls and early AMD). Therefore, this variation seems to mostly likely be AMD-related, although bearing a possible contribution from age. Altogether, the above analysis shows that the putative metabolite fingerprints listed in Table 2 are not significantly affected by differences in gender, smoking history or even age across patients’ groups, thus establishing that no group restrictions are necessary regarding these characteristics. This was also confirmed by covariance analysis of the main suggested fingerprint metabolites/resonances in Table 2 with gender proportion, smoking history, BMI, age and AMD status (Table 3). The consistently higher contributions of AMD severity (noted in bold) confirmed AMD as the primary origin of the metabolite changes observed. This is also supported by the fact that the role of comorbidities (S2 Table) was found to be negligible, as discussed below.

**Table 3:** Generalized linear regression results. Generalized linear regression coefficients obtained through modeling of metabolite variations as a function of gender proportion, smoking history, body-mass index (BMI), age and AMD status. F.A.: Fatty acids. Values in bold illustrate the higher contributions of AMD status for each metabolite variation, compared to confounders. Similar metabolite variations in the two cohorts are denoted by underline.

Compound	Gender proportion	Smoking history	BMI	Age	AMD status
**Coimbra cohort**					
Acetate	-4.0x10^-5^	2.1x10^-5^	-5.3x10^-6^	1.0x10^-6^	**1.1x10**^**-3**^
Acetoacetate	-3.8x10^-5^	-3.1x10^-5^	-3.1x10^-6^	7.3x10^-6^	**6.9x10**^**-4**^
Creatine	1.4x10^-4^	-1.8x10^-5^	4.2x10^-6^	-4.8x10^-6^	**1.6x10**^**-3**^
Dimethyl sulfone	-1.8x10^-5^	2.8x10^-5^	-4.8x10^-7^	7.5x10^-6^	**2.4x10**^**-4**^
HC = CH F.A.	7.6x10^-4^	2.9x10^-4^	1.2x10^-5^	-3.3x10^-5^	**1.5x10**^**-2**^
Histidine	1.3x10^-7^	-6.5x10^-6^	-1.3x10^-6^	3.1x10^-8^	**8.1x10**^**-4**^
β -hydroxybutyrate	-1.3x10^-5^	-4.6x10^-6^	-1.3x10^-6^	2.5x10^-6^	**4.6x10**^**-4**^
Pyruvate	-9.9x10^-5^	1.5x10^-5^	3.3x10^-6^	6.2x10^-7^	**9.3x10**^**-4**^
**Boston cohort**					
Alanine	-8.0x10^-5^	2.9x10^-5^	-2.0x10^-5^	-5.5x10^-6^	**4.2x10**^**-3**^
Glutamine	-4.6x10^-4^	-1.2x10^-4^	-8.5x10^-5^	3.2x10^-5^	**7.1x10**^**-3**^
Histidine	-2.3x10^-5^	4.0x10^-6^	-4.0x10^-6^	3.6x10^-6^	**5.1x10**^**-4**^
HC = CH F.A.	3.0x10^-4^	-1.7x10^-4^	5.5x10^-6^	-5.4x10^-5^	**1.4x10**^**-2**^

## Discussion

We present a cross-sectional study of two distinct cohorts of subjects with different severity stages of AMD and corresponding control groups. Using NMR metabolomics, we have observed small differences in the levels of some circulating metabolites (some amino acids and organic acids, dimethyl sulfone, lipid and protein moieties) between multiple AMD stages, in both cohorts. The potential confounding effects of gender, smoking history and age on these results were found to be negligible. To our knowledge, this study presents the first characterization of the different stages of AMD using NMR metabolomics, building up on a previous report [29] of MS metabolomics of the plasma of a small cohort of subjects with a single subtype of late AMD (“wet” AMD), compared to controls. The authors reported higher circulating levels of di- and tripeptides and modified amino acids in patients with AMD, along with lower levels of bile acids, histidine-arginine and tryptophan-phenylalanine, and metabolites related to vitamin-D metabolism. Our observation of changes in amino acids and protein levels in AMD patients is in broad agreement with the previously reported changes.

Interestingly, the metabolomic fingerprints of AMD suggested for the two cohorts presented both similarities and differences. The observed similarities in the variations of histidine, unsaturated fatty acids and protein levels suggest that such variations may be a global reflection of the disease, possibly transversal to different cohorts and, therefore, with potential value in contributing to the current knowledge of the biology of AMD. On the other hand, cohort differences in relation to variations in particular low-M_w_ compounds (e.g. glutamine, alanine, creatine, dimethyl sulfone, pyruvate) may reflect the potential importance of local nutritional and lifestyle effects on the suggested AMD metabolic fingerprints. Also of note are the differences observed between controls and subjects with early AMD, in both cohorts. These observations relate to two naturally age-matched groups (thus not affected by possible age effects on the metabolome) and may contribute importantly to the future definition of AMD biomarkers.

In any case, even though age was a potential major confounder in this study, differences in this parameter were found to only possibly affect plasmatic dimethyl sulfone levels, leaving the remaining fingerprint unaffected. Apart from other potential confounder contributions (gender and smoking habits), which were also found negligible, the possible effect of comorbidities had to be considered. Indeed, some comorbidities differed in proportion between intermediate and late AMD (S2 Table): heart, liver and rheumatologic diseases, in the Coimbra cohort; and kidney and neurologic diseases in the Boston cohort. However, in most cases only a few % of subjects were affected by these conditions (average 5%), thus rendering significant contributions unlikely. Heart disease in the Coimbra cohort may constitute an exception since about 23% of subjects with intermediate AMD were affected, compared to 6% of subjects with late AMD. It is, thus, possible that the metabolite changes noted from intermediate to late AMD in Coimbra may be at least partially due to heart disease, particularly regarding acetoacetate and β-hydroxybutyrate levels, since histidine variations (common to both cohorts) seem to constitute an AMD-related feature.

Acknowledged limitations of this study include its cross-sectional nature, which precludes the assessment of AMD time progression. Also of note is the self-reporting nature of the questionnaire used to assess demographics and prior medical history, which allows for the possibility of some degree of response bias. Despite representing the first complete analysis of AMD by NMR metabolomics, with the inclusion of more than 30 subjects in all study groups, higher sample sizes would be desirable in future validation studies. In addition, the important assessment of the influence of relevant confounders (assuming that their impact on plasma metabolome is independent of disease) should, in future studies, include the impacts of diet and genetic profiles on AMD metabolomic fingerprints. The study presented here benefits from the fact that it was prospectively designed to follow adequate standard operating procedures at all stages (metadata, samples and data collection), while ensuring that each individual underwent a complete ophthalmologic exam performed by a retina specialist. The latter aspect is particularly important since it circumvents the possible subjectivity of procedures relying on repositories or databases, which may lack in adequate phenotypic information, particularly regarding ophthalmic diseases.

## Conclusions

Our results demonstrate for the first time that plasma NMR metabolomics of patients with AMD detects small changes in the levels of selected amino acids and organic acids, as well as particular lipid moieties and protein levels. The changes observed are not very robust, but appear to be associated with the presence of the disease and its severity, rather than age, based on the identification of the particular age-dependent metabolite sets in each cohort. Other potential confounders (gender, smoking history, BMI, comorbidities) were also found not to affect the proposed AMD signatures significantly. Furthermore, the AMD-specific metabolite variations detected were found to partially differ between the two cohorts (Coimbra, Portugal and Boston, US), thus indicating that nutritional and other lifestyle habits may be determining the metabolic response in AMD in different regions. A particularly important result of this study is that, in each cohort, a subgroup of metabolite changes tended to differentiate the controls from the patients diagnosed with early AMD stage (namely acetate, creatine, dimethyl sulfone, cholesterol, HDL-choline and unsaturated fatty acids for Coimbra subjects, and albumin, histidine, glutamine and also unsaturated fatty acids for Boston subjects). This is an important observation that needs to be further investigated since it may contribute to the future definition of AMD biomarkers.

In summary, our results suggest that even though the overall metabolite changes detected in relation to AMD in both cohorts are of low magnitude and weak statistical relevance, they appear to be AMD-specific and should, therefore, be explored further in expanded cohorts and with methodologies targeting the metabolic domains of the specific compounds identified. This work has the potential to offer novel biomarkers for AMD, as well as to improve the current understanding of the pathogenesis of this blinding disease.

## Supporting information

S1 Appendix Questionnaire used in this study to assess demographics and prior medical history of each individual. (PDF)

S1 Fig Age and BMI histograms. Histograms of age and BMI distributions for controls and AMD patients for Coimbra and Boston cohorts: controls (^____^), early AMD patients (^**……**^), intermediate AMD patients (- - - -) and late AMD patients (-∙-∙-).
(TIFF)

S2 Fig Effect size plots for diffusion-edited spectra integrals. Plots of effect size (E.S.) for integrals measured in diffusion-edited spectra a) Coimbra and b) Boston cohorts. F.A.: fatty acids. Resonance list: *N*-acetyl-glycoproteins, δ 2.02–2.05; albumin-lysil groups, δ 2.92–3.02; protein NH region, δ 5.50–10.0; C18H cholesterol, δ 0.59–0.70; F.A. resonances: CH_3_ HDL, δ 0.79–0.85; CH_3_ LDL + VLDL, δ 0.85–0.91; (CH_2_)_n_ HDL, δ 1.18–1.25; (CH_2_)_n_ LDL+VLDL, δ 1.25–1.37; CH_2_CH_2_COOR, δ 1.45–1.62; CH_2_CH_2_CH = CH, δ 1.62–1.74; CH_2_CH = C, δ 1.90–2.02; CH_2_COOR, δ 2.17–2.26; C = CCH_2_C = C, δ 2.65–2.84; N(CH_3_)_3_ choline HDL, δ 3.19–3.21; N(CH_3_)_3_ choline LDL+VLDL, δ 3.23–3.26; CH_2_-N(CH_3_)_3_ choline, δ 3.62–3.68; Glyceryl C1,3H, δ 4.02–4.10; Glyceryl C1,3H’, δ 4.21–4.32; Glyceryl C2H, δ 5.13–5.21; HC = CH F.A. HDL, δ 5.24–5.28; HC = CH F.A. LDL+VLDL, δ 5.28–5.37. E.S. segments not intercepting the null E.S. line are considered as reflecting relevant variations (shaded rectangles).
(TIFF)

S1 Table Assignment of lipid NMR resonances. Assignment of lipid NMR resonances and corresponding lipid structures. Chemical shift ranges are indicated, as all resonances are broad and often structured. F.A.: fatty acids. R: alkyl group.
(DOCX)

S2 Table List of comorbidities found in the different subject groups considered. Comorbidities characterizing each of the subject groups with corresponding percentages and statistical relevance; p-values < 0.05 (Fisher Exact Test) are highlighted in bold; NA: non applicable.
(DOCX)

S3 Table Pairwise PLS-DA quality parameters. Pairwise PLS-DA quality parameters, Q^2^ (predictive power of pairwise model), classification rate (CR), % sensitivity (sens.) and % specificity (spec.), for PLS-DA models obtained with original (full) spectra and variable-selected spectra, for Coimbra and Boston cohorts and for CPMG and diffusion-edited spectra. Values in bold and underlined refer to best PLS-DA models.
(DOCX)
