# Supplementary material for: Human plasma metabolomics in age-related macular degeneration (AMD) using nuclear magnetic resonance spectroscopy
Source: PLoS One. 2017 May 18;12(5):e0177749. doi: 10.1371/journal.pone.0177749 (PMC5436712; doi:10.1371/journal.pone.0177749)
Supplement: S1 Appendix — (PDF) [file pone.0177749.s001.pdf]

Study title: Metabolomics, Genetics and Environment – a novel integrative approach to Age-Related Macular Degeneration

Medical and Ophthalmologic history

Version 1, 03/12/2014

STUDY ID: _________________ **Date: _________________**

1. DEMOGRAPHICS - For questions with boxes please fill just one box.

1.1 Gender ☐Male ☐Female **1.2 Race** ☐White ☐Black ☐Asian ☐Hispanic ☐Other

1.3 Occupation held most of life _________________

1.4 Number of **years working in this occupation** _________________

1.5 Are you currently ☐Working ☐ Retired

1.6 Highest scholar degree ☐Up to 4 years ☐ 5 to 9 years ☐ 10 to 12 years ☐ More than 12 years

1.7 Are you currently ☐Married/Unmarried couple ☐Divorce/ Separate ☐ Single ☐ Widower

2. HABITS

2.1 Regarding smoking, are you currently a

☐ Non-smoker (i.e: never been a smoker)

☐ Smoker

☐ Ex-smoker (i.e: used to be a smoker, but not smoking anymore)

2.1.1 If your answer was smoker:

At what age did you start smoking: _______ (years)

How many cigarettes per day do you smoke? ___

2.2.2 If your answer was ex-smoker:

At what age did you start smoking: _______ (years)

At what age did you stop smoking: _______ (years)

How many cigarettes per day do you used to smoke? ___

3. MEDICAL HISTORY

3.1 Please fill the table below about your **current or past medical conditions**:

Condition	Has a doctor ever told you that you have any of the following conditions?	When was it diagnosed?	Other
Diabetes	☐ No ☐ Yes ☐ I don't know	(year of diagnosis)	Which medication are you currently taking fo diabetes? ☐ Pills ☐ Insulin ☐None ☐ I don't know
Hypertension	☐ No ☐ Yes ☐ I don't know	(year of diagnosis)	
High cholesterol	☐ No ☐ Yes ☐ I don't know	(year of diagnosis)	
High triglycerides	☐ No ☐ Yes ☐ I don't know	(year of diagnosis)	
Congestive heart failure	☐ No ☐ Yes ☐ I don't know	(year of diagnosis)	
NYHA functional class	☐ I ☐ II ☐ III ☐ IV		*I – Ordinary physical activity does not cause undue dyspnea or fatigue<br>II – Ordinary physical activity results in dyspnea or fatigue<br>III – Less than ordinary activity causes dyspnea or fatigue but comfortable at rest<br>IV – Dyspnea or fatigue present even at rest, increasing if any physical activity is undertaken
Angina pectoris*	☐ No ☐ Yes ☐ I don't know	(year of diagnosis)	*Pain, heaviness or pressure in chest or upper body when walking quickly or uphill, which subdues when at rest
Heart attack (myocardial infarction)	☐ No ☐ Yes ☐ I don't know	(year of diagnosis)	
Heart surgery, bypass or vascular surgery	☐ No ☐ Yes ☐ I don't know	(year of diagnosis)	Type of surgery: ___________________________________
Blood or clotting disorders	☐ No ☐ Yes ☐ I don't know	(year of diagnosis)	Type of blood or clotting disorder: ___________________________________
Stroke or transient ischemic attack	☐ No ☐ Yes ☐ I don't know	(year of diagnosis)	
Kidney disease	☐ No ☐ Yes ☐ I don't know	(year of diagnosis)	What type of kidney disease do you have? ___________________________________ Have you been submitted to a kidney transplant? ☐ No ☐ Yes

			Are you currently doing dialysis?<br>☐ No ☐ Yes
.iver disease	☐ No ☐ Yes ☐ I don't know	(year of diagnosis)	What type of liver disease do you have?<br>_________________________________
Vigraine	☐ No ☐ Yes ☐ I don't know	(year of diagnosis)	
3rain trauma	☐ No ☐ Yes ☐ I don't know	(year of diagnosis)	
Epilepsy	☐ No ☐ Yes ☐ I don't know	(year of diagnosis)	
Parkinson disease	☐ No ☐ Yes ☐ I don't know	(year of diagnosis)	
Any form of dementia	☐ No ☐ Yes ☐ I don't know	(year of diagnosis)	
Other neurologic diseases	☐ No ☐ Yes ☐ I don't know	(year of diagnosis)	If yes, which type of neurologic disease?<br>__________________________________
Cancer	Have you ever had a diagnosis of cancer?<br>☐ No ☐ Yes ☐ I don't know	(year of diagnosis)	If yes, which type of cancer do you have diagnosed?<br>_________________________________<br>Are you currently undergoing treatment for the cancer?<br>☐ No ☐ Yes ☐ I don't know<br>If yes, which type of treatment?<br>☐Chemotherapy ☐ Radiotherapy<br>☐ Hormonal therapy ☐ I don't know ☐ None of this<br>When (year) was your cancer considered in remission?<br>_________________
Psoriasis/ eczema	☐ No ☐ Yes ☐ I don't know	(year of diagnosis)	

Rheumatoid arthritis	☐ No ☐ Yes ☐ I don't know	(year of diagnosis)	
Systemic lupus erythematous	☐ No ☐ Yes ☐ I don't know	(year of diagnosis)	
Multiple sclerosis	☐ No ☐ Yes ☐ I don't know	(year of diagnosis)	
Thyroid disease	☐ No ☐ Yes ☐ I don't know	(year of diagnosis)	

3.2 If you are a woman, please answer to the following questions. If you are a man, please skip to question 4.

3.2.1 Did you already reach the menopause? ☐No ☐Yes ☐ I don't know

If you already reach the menopause, please reply also to these questions:

At what age did you reach the menopause? ________________

Are you currently doing hormonal therapy? ☐No ☐Yes

If yes, when did you start it? ____________(mm/dd/yy)

Have you done hormonal therapy in the past? ☐No ☐Yes

If yes, when did you start it? ________________ (mm/dd/yy)

If yes, when did you stop it? ________________ (mm/dd/yy)

4. OPHTHALMOLOGICAL HISTORY

4.1 Do you currently use glasses or contact lens? ☐No ☐Yes ☐I don't know

4.1.1 If yes, do you know your current refraction? If yes, please write it down below:

Right eye ______________________ Left eye_____________________________

4.2 Do you have any known ocular diseases? ☐No ☐Yes ☐I don't know

If yes, please fill the table below

Which type of ocular disease do you have, e.g., glaucoma, cataracts, dry eye?	Which eye is affected?	When was it diagnosed?

	☐Right ☐Left ☐Both ☐I don't know	(year)
	☐Right ☐Left ☐Both ☐I don't know	(year)
	☐Right ☐Left ☐Both ☐I don't know	(year)
	☐Right ☐Left ☐Both ☐I don't know	(year)
	☐Right ☐Left ☐Both ☐I don't know	(year)
	☐Right ☐Left ☐Both ☐I don't know	(year)

4.3 Are you currently using any eye drops? ☐No ☐Yes ☐I don't know

If yes, please fill the table below with the names and details about all the eye drops that you are currently using.

Name of the eye drop	In which eye are you using the eye drop?	For how long have you been using the eye drop?	How many times per day are you applying this eye drop?
	☐Right ☐Left ☐Both ☐I don't know	(number of months)	
	☐Right ☐Left ☐Both ☐I don't know	(number of months)	
	☐Right ☐Left ☐Both ☐I don't know	(number of months)	
	☐Right ☐Left ☐Both ☐I don't know	(number of months)	

4.4 Have you had any eye surgeries? ☐No ☐Yes ☐I don't know

If yes, please fill the table below

Which type of eye surgery have you had?	Which was the operated eye?	When was the surgery performed?
	☐Right ☐Left ☐Both ☐I don't know	(mm/dd/yy)
	☐Right ☐Left ☐Both ☐I don't know	(mm/dd/yy)
	☐Right ☐Left ☐Both ☐I don't know	(mm/dd/yy)

4.5 Have you had any laser treatments in the eye? ☐No ☐Yes ☐I don't know

If yes, please fill the table below

Type of laser	Did you receive this laser treatment in the past?	Which was the treated eye?	When was the laser performed?
Refractive laser (ie: PRK, LASIK)	☐ No ☐ Yes ☐ I don't know	☐Right ☐Left ☐Both ☐I don't know	(yea[illegible]<br>(!) If both eyes pleas[illegible] introduce the mos[illegible] recent dat[illegible]

Photodynamic therapy ("PDT" or "Visudyne")	☐ No ☐ Yes ☐ I don't know	☐Right ☐Left ☐Both ☐I don't know	(yea[illegible]<br>(!) If both eyes pleas[illegible] introduce the mos[illegible] recent dat[illegible]
Retinal laser (photocoagulation) therapy	☐ No ☐ Yes ☐ I don't know	☐Right ☐Left ☐Both ☐I don't know	(mm/dd/y[illegible]<br>(!) If several session[illegible] please introduce th[illegible]
YAG capsulotomy (ie: laser to "clean" the lens after cataract surgery)	☐ No ☐ Yes ☐ I don't know	☐Right ☐Left ☐Both ☐I don't know	(yea[illegible]<br>(!) If both eyes pleas[illegible] introduce the mos[illegible] recent dat[illegible]

4.6 Have you had injections in the eye? ☐No ☐Yes ☐I don't know

If yes:

4.6.1 Which type of injections did you receive?

☐Lucentis (Ranibizumab) ☐Avastin (Bevacizumab) ☐Eylea (Aflibercept) ☐I don't know

4.6.2 Which eye received the injections? ☐Right ☐Left ☐Both ☐I don't know

4.6.3 How many injections did you already received?

Right eye: __________ Left eye: __________ ☐I don't know

4.6.4 When was the day of your last injection?

Right eye: __________ (mm/dd/yy) Left eye: __________ (mm/dd/yy) ☐I don't know

5. CURRENT SYSTEMIC MEDICATION

5.1 Vitamins

5.1.1 Are you currently taking vitamin supplements? ☐Yes ☐No ☐I don't know

If yes, please fill this table

Name of vitamin supplement	Dose	Frequency	How many months ago did you start?

If you replied yes to the two previous questions, please skip to question 5.1.2

5.1.2 Have you taken vitamin supplements in the past? ☐Yes ☐No ☐I don't know

If yes, please fill this table

Name of vitamin supplement	Dose	Frequency	Year start	Year end

5.2 Other medications

Please fill this table with all the remaining medication that you are currently taking?

Name of medication	Dose	Frequency	Date start (mm/dd/yy)	Reason for taking the medication

6. Other

6.1 Dou you have any direct relatives with Age-Related Macular Degeneration? ☐No ☐Yes ☐I don't know

If yes, please fill the boxes to all that apply:

☐ Father ☐ Mother ☐ Aunt or uncle ☐ Brother or sister ☐ Son or daughter

Do you know which type of AMD these relatives have (wet/dry)?
____ (please specify for each relative)

6.2 Dou you have any direct relatives with neurologic or psychiatric diseases? ☐No ☐Yes ☐I don't know

If yes, please fill the boxes to all that apply:

☐ Father ☐ Mother ☐ Aunt or uncle ☐ Brother or sister ☐ Son or daughter

Please specify which type of neurological or psychiatric disease for each of the above mentioned relatives:

____

____

____
