# Supplementary figures and images for: Human plasma metabolomics in age-related macular degeneration (AMD) using nuclear magnetic resonance spectroscopy
Source: PLoS One. 2017 May 18;12(5):e0177749. doi: 10.1371/journal.pone.0177749 (PMC5436712; doi:10.1371/journal.pone.0177749)

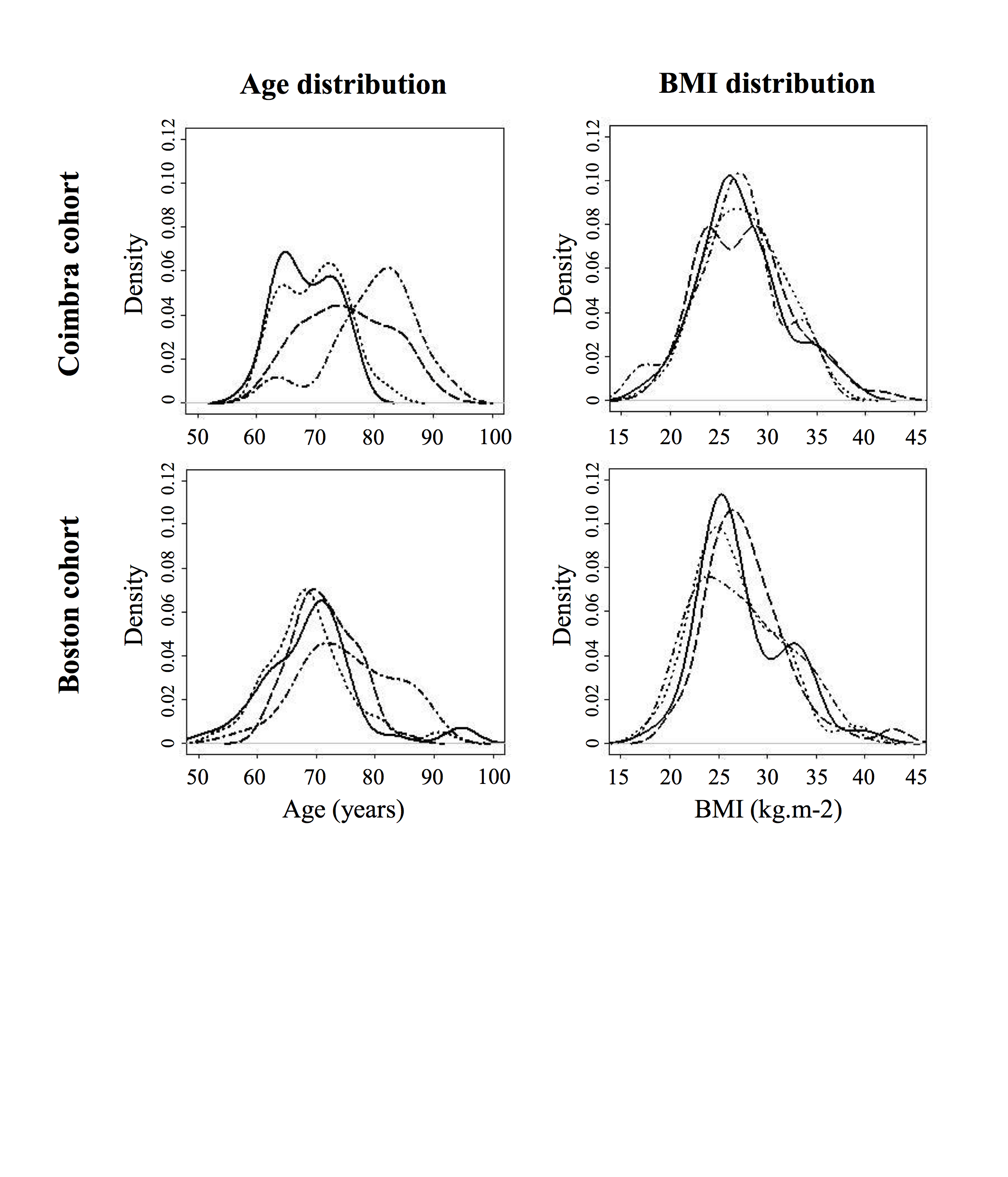

Supplement: S1 Fig — Histograms of age and BMI distributions for controls and AMD patients for Coimbra and Boston cohorts: controls (____), early AMD patients (……), intermediate AMD patients (- - - -) and late AMD patients (-∙-∙-). (TIFF) [file pone.0177749.s002.tiff]

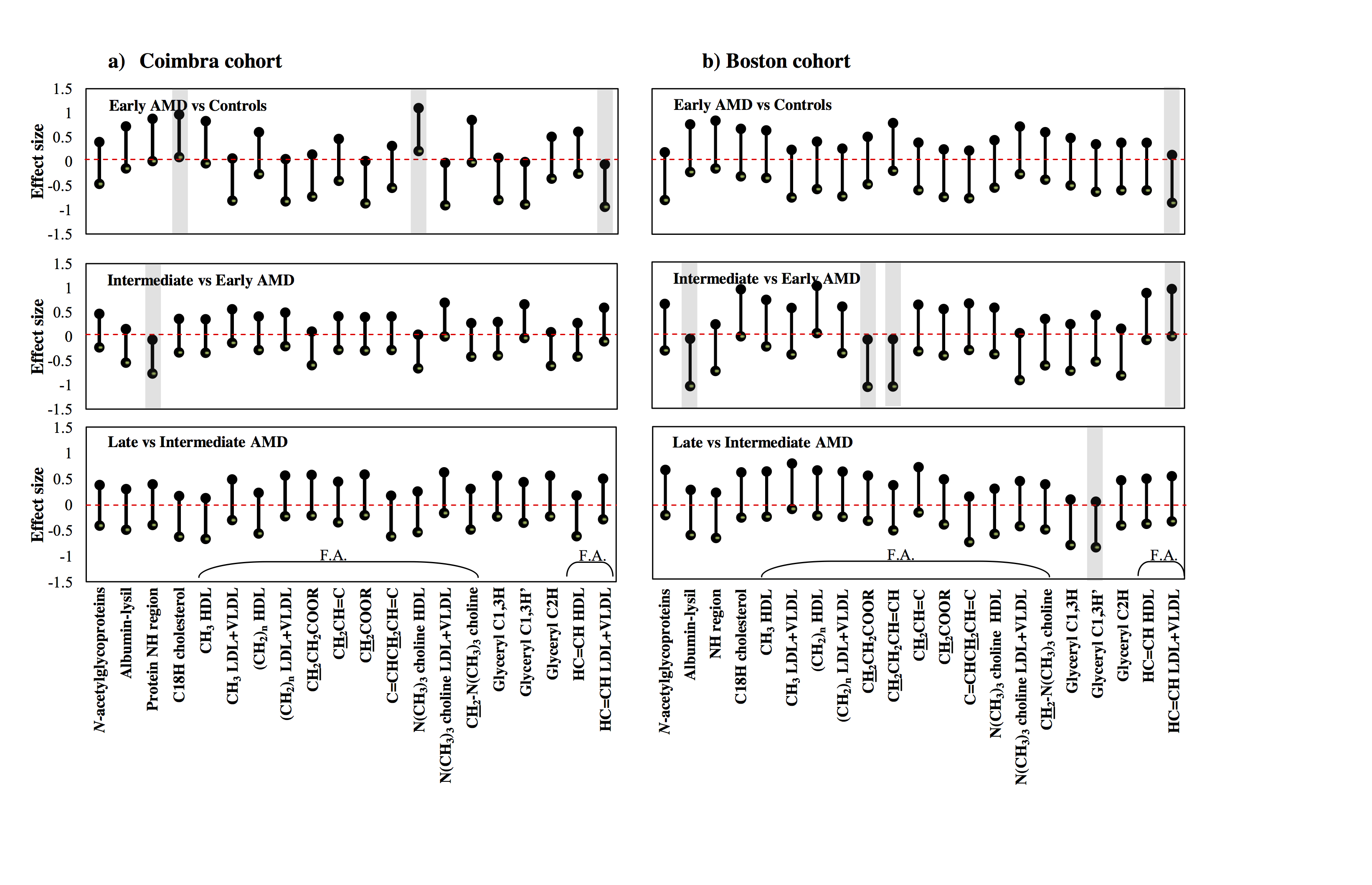

Supplement: S2 Fig — Plots of effect size (E.S.) for integrals measured in diffusion-edited spectra a) Coimbra and b) Boston cohorts. F.A.: fatty acids. Resonance list: N-acetyl-glycoproteins, δ 2.02–2.05; albumin-lysil groups, δ 2.92–3.02; protein NH region, δ 5.50–10.0; C18H cholesterol, δ 0.59–0.70; F.A. resonances: CH3 HDL, δ 0.79–0.85; CH3 LDL + VLDL, δ 0.85–0.91; (CH2)n HDL, δ 1.18–1.25; (CH2)n LDL+VLDL, δ 1.25–1.37; CH2CH2COOR, δ 1.45–1.62; CH2CH2CH = CH, δ 1.62–1.74; CH2CH = C, δ 1.90–2.02; CH2COOR, δ 2.17–2.26; C = CCH2C = C, δ 2.65–2.84; N(CH3)3 choline HDL, δ 3.19–3.21; N(CH3)3 choline LDL+VLDL, δ 3.23–3.26; CH2-N(CH3)3 choline, δ 3.62–3.68; Glyceryl C1,3H, δ 4.02–4.10; Glyceryl C1,3H’, δ 4.21–4.32; Glyceryl C2H, δ 5.13–5.21; HC = CH F.A. HDL, δ 5.24–5.28; HC = CH F.A. LDL+VLDL, δ 5.28–5.37. E.S. segments not intercepting the null E.S. line are considered as reflecting relevant variations (shaded rectangles). (TIFF) [file pone.0177749.s003.tiff]
