# Supplementary material for: Human plasma metabolomics in age-related macular degeneration (AMD) using nuclear magnetic resonance spectroscopy
Source: PLoS One. 2017 May 18;12(5):e0177749. doi: 10.1371/journal.pone.0177749 (PMC5436712; doi:10.1371/journal.pone.0177749)
Supplement: S1 Table — Assignment of lipid NMR resonances and corresponding lipid structures. Chemical shift ranges are indicated, as all resonances are broad and often structured. F.A.: fatty acids. R: alkyl group. (DOCX) [file pone.0177749.s004.docx]

|  | **Table S1** |  | |
| --- | --- | --- | --- |
| **Lipid moiety** | **Chemical shift range (δ/ppm)** | **Protons assigned** | |
| C18H Cholesterol | 0.60-0.70 | 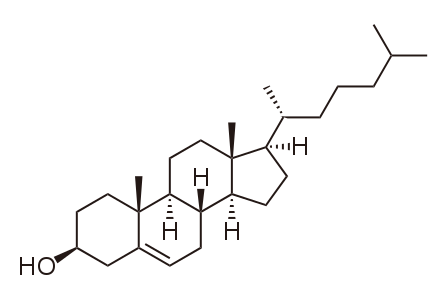 |  |
| CH_3_ fatty acids | 0.79-0.91 | 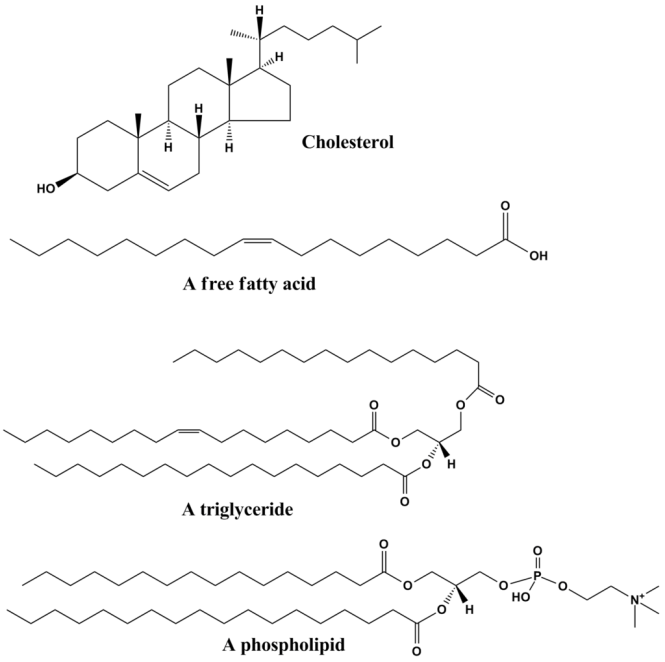OR |  |
| (CH_2_)_n_ fatty acids | 1.18-1.37 | 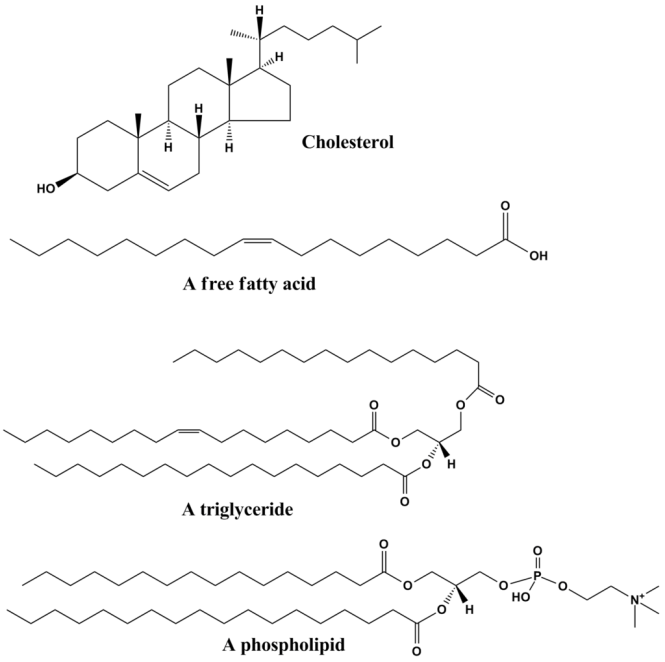OR |  |
| CH_2_CH_2_CO fatty acids | 1.45-1.62 | 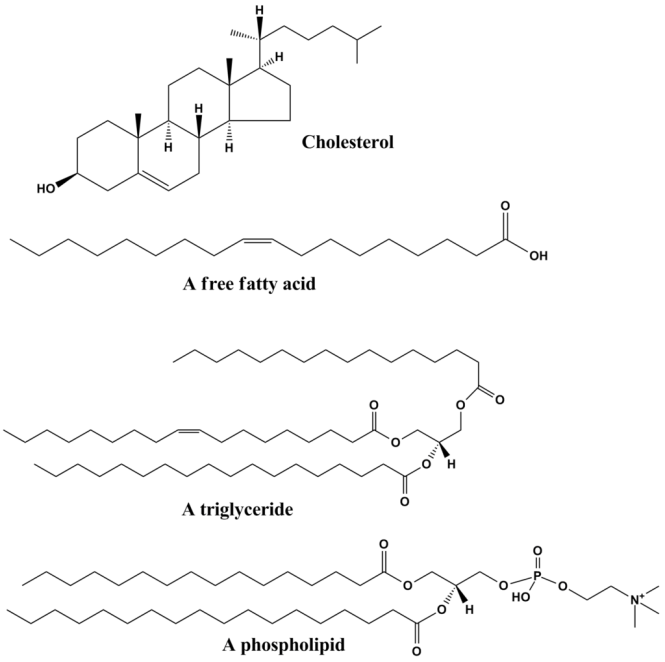OR |  |
| CH_2_CH_2_CH=C fatty acids | 1.62-1.74 | 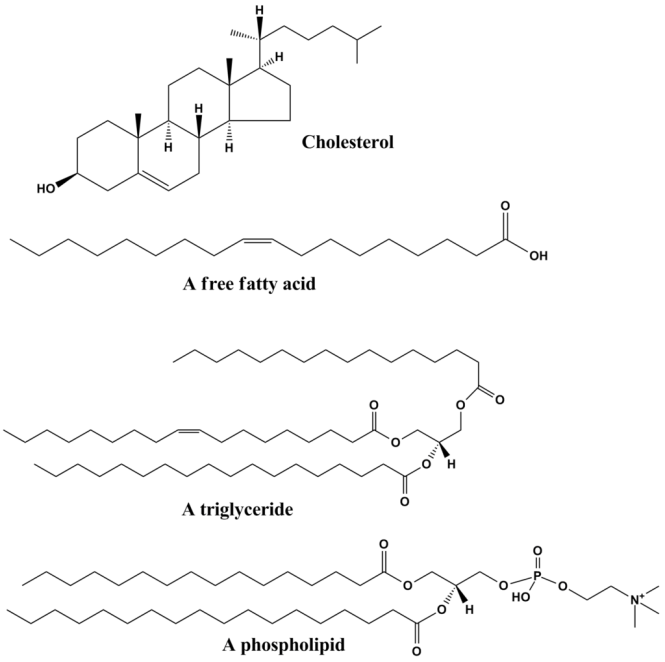OR |  |
| CH_2_CH=C fatty acids | 1.90-2.02 | 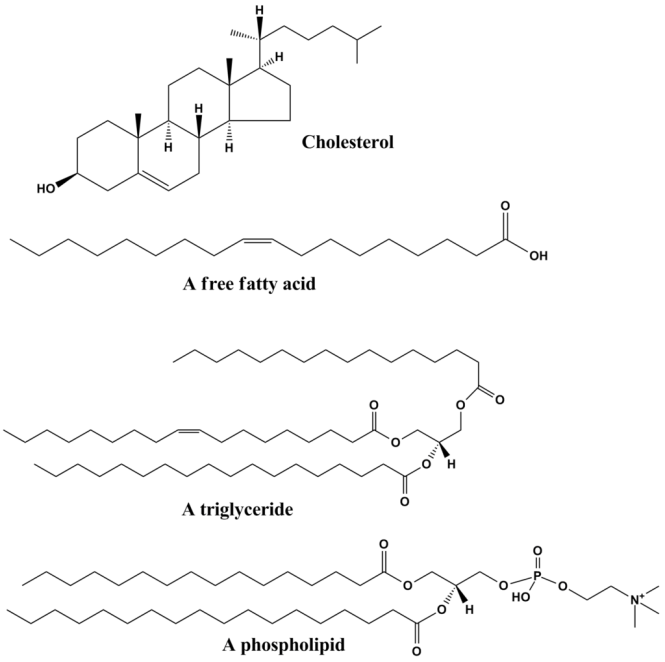OR |  |
| CH_2_CO fatty acids | 2.17-2.26 | 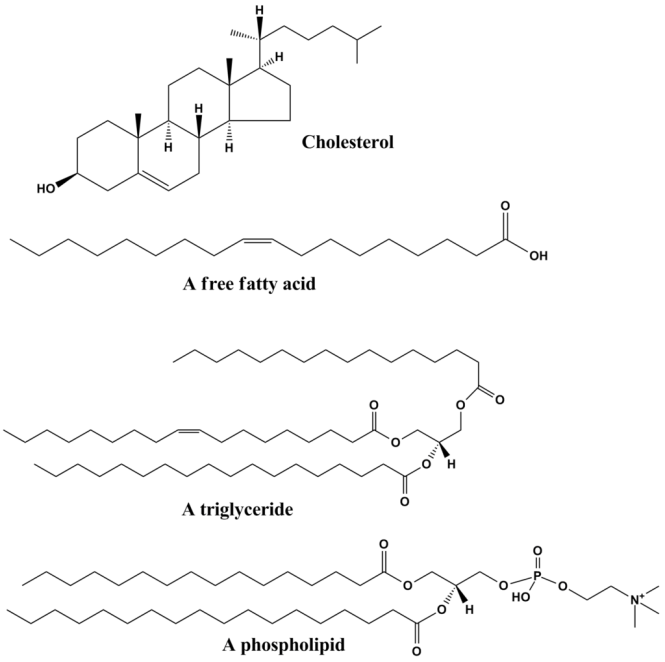OR |  |
| CH=CH_2_CH=C fatty acids | 2.65-2.84 | 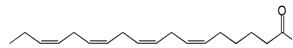OR |  |
| N(CH_3_)_3_ choline | 3.19-3.21 | 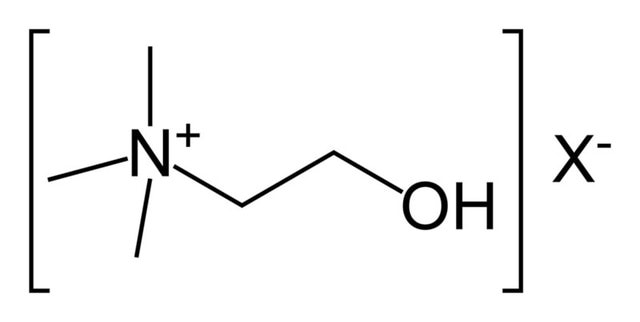  OR |  |
| CH_2_-N(CH_3_)_3_ choline | 3.62-3.68 | 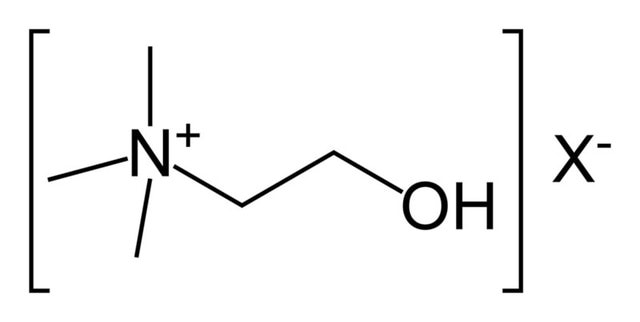  OR |  |
| Glyceryl C1,3H | 4.02-4.10  OR | OR  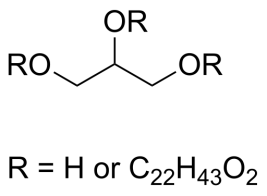  OR |  |
| Glyceryl C1,3H’ | 4.21-4.32  OR | OR  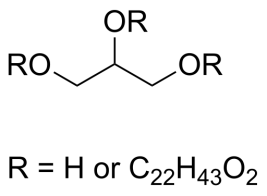  OR |  |
| Glyceryl C2H | 5.13-5.21 | OR  OR  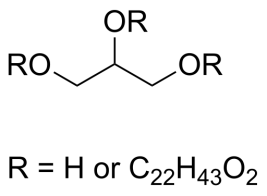  OR |  |
| HC=CH fatty acids | 5.24-5.37 | 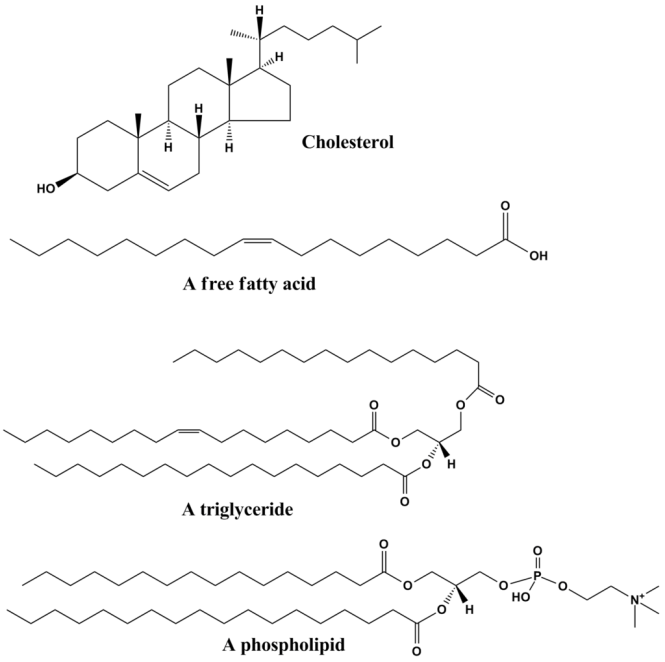OR |  |
