# Supplementary material for: Human plasma metabolomics in age-related macular degeneration (AMD) using nuclear magnetic resonance spectroscopy
Source: PLoS One. 2017 May 18;12(5):e0177749. doi: 10.1371/journal.pone.0177749 (PMC5436712; doi:10.1371/journal.pone.0177749)
Supplement: S2 Table — Comorbidities characterizing each of the subject groups with corresponding percentages and statistical relevance; p-values < 0.05 (Fisher Exact Test) are highlighted in bold; NA: non applicable. (DOCX) [file pone.0177749.s005.docx]

|  | **Coimbra** | | | | **Boston** | | | |
| --- | --- | --- | --- | --- | --- | --- | --- | --- |
|  | **Control**  n (%) | **Early AMD**  n (%), p-value | **Int. AMD**  n (%), p-value | **Late AMD**  n (%), p-value | **Control**  n (%) | **Early AMD**  n (%), p-value | **Int. AMD**  n (%), p-value | **Late AMD**  n (%), p-value |
| Total | 42 (17.3) | 45 (18.5), NA | 124 (51.0), NA | 32 (13.2), NA | 40 (26.1) | 30 (19.6), NA | 45 (29.4), NA | 38 (24.8), NA |
| Hypertension | 21 (50.0) | 23 (51.1), 0.918 | 71 (57.3), 0.489 | 19 (59.4), 0.829 | 14 (35) | 7 (23.3), 0.430 | 16 (35.6), 0.313 | 20 (52.6), 0.128 |
| Dyslipidemia | 19 (45.2) | 25 (55.6), 0.394 | 54 (43.6), 0.222 | 19 (59.4), 0.117 | 15 (37.5) | 9 (30), 0.614 | 19 (42.2), 0.336 | 15 (39.5), 0.826 |
| Heart disease | 8 (19.1) | 7 (15.6), 0.799 | 29 (23.4), 0.395 | 2 (6.3), **0.044** | 2 (5) | 6 (20), 0.066 | 7 (15.6), 0.757 | 6 (15.8), 0.977 |
| Blood disease | 1 (2.4) | 0 (0), 0.483 | 5 (4.0), 0.326 | 3 (9.4), 0.362 | 5 (12.5) | 3 (10), 0.745 | 4 (8.9), 0.871 | 2 (5.3), 0.683 |
| Stroke | 0 (0) | 1 (2.2), 0.331 | 8 (6.5), 0.447 | 3 (9.4), 0.697 | 5 (12.5) | 0 (0), 0.064 | 2 (4.4), 0.514 | 2 (5.3), 0.862 |
| Renal disease | 1 (2.4) | 2 (4.4), 0.598 | 6 (4.8), 0.915 | 1 (3.2), 0.676 | 2 (5) | 2 (6.7), 0.766 | 0 (0), 0.157 | 5 (13.2), **0.017** |
| Liver disease | 0 (0) | 1 (2.2), 0.331 | 1 (0.8), 0.463 | 3 (9.4), **0.027** | 2 (5) | 1 (3.3), 0.733 | 1 (2.2), 0.770 | 0 (0), 0.355 |
| Cancer history | 5 (11.9) | 4 (8.9), 0.733 | 11 (8.9), 1.000 | 2 (6.3), 0.632 | 12 (30) | 9 (30), 1.000 | 21 (46.7), 0.229 | 12 (31.6), 0.183 |
| Rheumatic disease | 0 (0) | 1 (2.2), 0.331 | 1 (0.8), 0.463 | 2 (9.4), **0.027** | 4 (10) | 4 (13.3), 0.664 | 10 (22.2), 0.382 | 9 (23.7), 0.874 |
| Neurologic disease | 9 (21.4) | 11 (24.4), 0.802 | 19 (15.3), 0.178 | 6 (18.8), 0.599 | 3 (7.5) | 2 (6.7), 0.893 | 5 (11.1), 0.695 | 0 (0), 0.059 |

**Table S2**
