# Supplementary material for: Human plasma metabolomics in age-related macular degeneration (AMD) using nuclear magnetic resonance spectroscopy
Source: PLoS One. 2017 May 18;12(5):e0177749. doi: 10.1371/journal.pone.0177749 (PMC5436712; doi:10.1371/journal.pone.0177749)
Supplement: S3 Table — Pairwise PLS-DA quality parameters, Q2 (predictive power of pairwise model), classification rate (CR), % sensitivity (sens.) and % specificity (spec.), for PLS-DA models obtained with original (full) spectra and variable-selected spectra, for Coimbra and Boston cohorts and for CPMG and diffusion-edited spectra. Values in bold and underlined refer to best PLS-DA models. (DOCX) [file pone.0177749.s006.docx]

**Table S3**

|  |  | **CPMG spectra** | | | | | | | | | | | | **Diffusion-edited spectra** | | | | | | | | | | | | | | | | | |  |
| --- | --- | --- | --- | --- | --- | --- | --- | --- | --- | --- | --- | --- | --- | --- | --- | --- | --- | --- | --- | --- | --- | --- | --- | --- | --- | --- | --- | --- | --- | --- | --- | --- |
|  |  | **Original spectra** | | | | | **Variable-selected spectra** | | | | | | | **Original spectra** | | | | | | | | **Variable-selected spectra** | | | | | | | | | |  |
|  |  | **Q^2^** | **%**  **CR** | | **%**  **Sens** | **%**  **Spec** | **% data** | **Q^2^** | **%**  **CR** | | **%**  **Sens** | | **%**  **Spec** | | **Q^2^** | | **%**  **CR** | | **%**  **Sens** | | **%**  **Spec** | | **% data** | | **Q^2^** | | **%**  **CR** | | **%**  **Sens** | | **%**  **Spec** | |
| **Coimbra** | | | | | | | | | | | | | | | | | | | | | | | | | | | | | | | |  |
| **AMD evolution** | Early *vs* Cont. | 0.06 | | 64 | 63 | 65 | 38.4 | 0.13 | 64 | 63 | | 65 | | -0.02 | | 60 | | 58 | | 61 | | 33.5 | | 0.15 | | 71 | | 85 | | 31 | |  |
|  | Int. *vs* Early | -0.02 | | 70 | 90 | 17 | 10.3 | 0.11 | 74 | 91 | | 25 | | 0.09 | | 73 | | 100 | | 0 | | 36.7 | | 0.12 | | 75 | | 94 | | 24 | |  |
|  | Late *vs* Int. | 0.034 | | 77 | 18 | 93 | 37.4 | 0.006 | 80 | 8 | | 99 | | -0.04 | | 79 | | 0 | | 100 | | 11.9 | | 0.05 | | 79 | | 2 | | 99 | |  |
| **Others comparisons** | Int. AMD *vs* Cont. | 0.10 | | 73 | 94 | 11 | 34.3 | 0.20 | 71 | 85 | | 31 | | -0.01 | | 74 | | 99 | | 0 | | 28.8 | | 0.10 | | 71 | | 98 | | 4 | |  |
|  | Late AMD *vs* Cont. | 0.30 | | 70 | 53 | 82 | 35.6 | **0.35** | **75** | **60** | | **87** | | 0.21 | | 46 | | 29 | | 60 | | 20.1 | | 0.24 | | 93 | | 87 | | 98 | |  |
|  | Late *vs* Early AMD | 0.004 | | 57 | 40 | 69 | 20.9 | **0.34** | **87** | **80** | | **91** | | 0.20 | | 53 | | 10 | | 85 | | 20.2 | | **0.32** | | **58** | | **21** | | **84** | |  |
| **Boston** | | | | | | | | | | | | | | | | | | | | | | | | | | | | | | | |  |
| **AMD evolution** | Early *vs* Cont. | 0.18 | 49 | | 39 | 57 | 17.3 | **0.54** | **97** | | **95** | | **98** | | 0.08 | | 59 | | 44 | | 71 | | 12.4 | | **0.50** | | **84** | | **76** | | **90** | |
|  | Int. *vs* Early | **0.32** | **69** | | **80** | **53** | 22.2 | **0.49** | **85** | | **90** | | **77** | | 0.20 | | 71 | | 75 | | 65 | | 39.9 | | 0.28 | | 75 | | 78 | | 71 | |
|  | Late *vs* Int. | -0.15 | 61 | | 67 | 54 | 31.2 | **0.32** | **77** | | **83** | | **71** | | 0.01 | | 63 | | 73 | | 51 | | 24.1 | | 0.28 | | 90 | | 90 | | 90 | |
| **Others comparisons** | Int. AMD *vs* Cont. | 0.14 | 49 | | 54 | 44 | 19.6 | 0.35 | 47 | | 0 | | 100 | | 0.10 | | 72 | | 61 | | 72 | | 38.5 | | **0.39** | | **70** | | **68** | | **73** | |
|  | Late AMD *vs* Cont. | 0.04 | 61 | | 65 | 73 | 27.6 | **0.41** | **80** | | **74** | | **85** | | 0.26 | | 71 | | 61 | | 81 | | 35.8 | | **0.35** | | **76** | | **75** | | **78** | |
|  | Late *vs* Early AMD | 0.09 | 50 | | 60 | 38 | 27.1 | **0.40** | **72** | | **87** | | **75** | | **0.39** | | **79** | | **78** | | **79** | | 33.4 | | **0.47** | | **78** | | **76** | | **81** | |
